# Lipid Nanoparticle Inclusion Prevents Capsaicin-Induced TRPV1 Defunctionalization

**DOI:** 10.3390/pharmaceutics12040339

**Published:** 2020-04-10

**Authors:** Carmelo Puglia, Debora Santonocito, Angela Bonaccorso, Teresa Musumeci, Barbara Ruozi, Rosario Pignatello, Claudia Carbone, Carmela Parenti, Santina Chiechio

**Affiliations:** 1 Dipartimento di Scienze del Farmaco, Università di Catania, 95100 Catania, Italy; debora.santonocito@outlook.it (D.S.); angela.bonaccorso87@gmail.com (A.B.); tmusumec@unict.it (T.M.); r.pignatello@unict.it (R.P.); ccarbone@unict.it (C.C.); cparenti@unict.it (C.P.); chiechio@unict.it (S.C.); 2 Dipartimento di Scienze della Vita, Università di Modena e Reggio Emilia, 41100 Modena, Italy; barbara.ruozi@unimore.it; 3 Oasi Research Institute-IRCCS, 94018 Troina, Italy

**Keywords:** capsaicin, TRPV1 agonist, lipid nanocarrier (LN), atomic force microscopy (AFM), in vitro release, spontaneous pain, western blot analysis

## Abstract

Background: Capsaicin (CPS) is a highly selective agonist of the transient receptor potential vanilloid type 1 (TRPV1) with a nanomolar affinity. High doses or prolonged exposure to CPS induces TRPV1 defunctionalization and, although this effect is currently used for the treatment of thermal hyperalgesia in chronic pain conditions, it is responsible of detrimental effects, such as denervation of sensory fibers. The aim of the present study was to formulate CPS loaded lipid nanocarriers (CPS-LN) in order to optimize CPS release, thus preventing TRPV1 internalization and degradation. Methods: CPS-LNs were formulated and characterized by in vitro studies. The activation of TRPV1 receptors after CPS-LN administration was evaluated by measuring spontaneous pain that was induced by local injection into the plantar surface of the mouse hind-paw. Moreover, the expression of TRPV1 in the skin was evaluated by western blot analysis in CPS-LN injected mice and then compared to a standard CPS solution (CPS-STD). Results: CPS inclusion in LN induced a lower pain response when compared to CPS-STD; further, it prevented TRPV1 down-regulation in the skin, while CPS-STD induced a significant reduction of TRPV1 expression. Conclusions: Drug encapsulation in lipid nanoparticles produced an optimization of CPS release, thus reducing mice pain behavior and avoiding the effects that are caused by TRPV1 defunctionalization related to a prolonged activation of this receptor.

## 1. Introduction

Capsaicin (CPS), which is the pungent ingredient of hot peppers, is a highly selective agonist of the transient receptor potential vanilloid type 1 (TRPV1) with nanomolar affinity [1]. The capsaicin TRPV1 receptor is a non-selective ligand-gated cation channel that formed by six membrane-spanning domains assembled as homo or hetero-tetramers activated by heat, low pH, and endogenous lipids [2,3,4]. TRPV1 receptors are widely expressed in areas that are involved in pain transmission, both in the central and peripheral nervous system [5,6]. In the brain, TRPV1 receptors are also involved in the modulation of synaptic plasticity as well as in epilepsy and cognition [7]. Within the peripheral nervous system, the TRPV1 receptors are located in sensory ganglia and in small sensory C and Aδ fibers [5,8], thus representing a promising target for the modulation of pain sensitivity [9,10]. In this context, the up- or down-regulation of TRPV1 receptors has been shown. In particular, the up-regulation of TRPV1 expression occurs during inflammation and in pathological pain states, such as neuropathic pain [11]. On the contrary, a down-regulation of the TRPV1 expression has been observed in neuropathic pain that is caused by injury [8]. TRPV1 receptors that are expressed in nociceptive sensory nerves (C and Aδ fibers) are targeted by topical capsaicin formulations, such as creams, lotions, or patches used for the management of neuropathic pain [12,13]. Upon exposure to CPS, TRPV1 receptors might undergo phosphorylation/dephosphorylation processes that contribute to phosphorylation-mediated TRPV1 desensitization or tachyphylaxis, a mechanism that accounts for acute TRPV1 desensitization. Moreover, high doses or prolonged exposure to CPS induces TRPV1 endocytosis and lysosomal degradation, resulting in a loss of function known as “nociceptor defunctionalization”. TRPV1 defunctionalization is also characterized by a reversible reduction in intra-epidermal sensory nerve fibers, a mechanism that contributes to long-term nociceptors desensitization [14,15]. Additionally, CPS can induce TRPV1 internalization followed by nerve degeneration after local and systemic high dose injection [16,17,18].

Although CPS-induced defunctionalization of TRPV1 receptors have been used for the treatment of thermal hyperalgesia in chronic pain conditions, evidence points to a physiological role of TRPV1 receptors and detrimental effects have been associated after long-term blockade or the down-regulation of these receptors. [19]. Therefore, it results in noteworthy interest the formulation of a drug delivery system able to produce a CPS slow release, thus preventing TRPV1 internalization and degradation.

In this context, liposomes and lipid nanoparticles (LN) have become increasingly interesting for scientists due to their potential to overcome drug delivery challenges [20,21]. The latter, in particular, developed in the last decade as alternative systems to existing traditional carriers, possess a great potential, and have generated a large interest in the industrial and academic worlds. In fact, they have been proposed and investigated for many different applications through all administration routes [22].

In this work, we sought to investigate whether a controlled release of CPS obtained by the inclusion of the drug in lipid nanoparticles might improve its pharmacokinetic profile, thus preventing the long-term down-regulation of TRPV1 and preserving the physiologic role of this receptors. 

To this aim, CPS-loaded lipid nanoparticles (CPS-LN) were formulated and characterized by in vitro studies. The activation of TRPV1 receptors after CPS-LN administration was evaluated by measuring spontaneous pain induced by local injection into the plantar surface of the mouse hind-paw. Moreover, the expression of TRPV1 in the skin was evaluated by western blot analysis in CPS-LN injected mice and then compared to a standard CPS solution.

## 2. Materials and Methods 

### 2.1. Materials

Capsaicin (CPS) was provided from Sigma–Aldrich (St. Louis, MO, USA). Softisan© 100 (Hydrogenated Coco-Glycerides) was a gift of Sasol (Witten, Germany). Tween 80 was provided from Polichimica S.r.l (Bologna, Italy). HPLC grade solvents and water were purchased from Carlo Erba reagents (CarloErba, Milan, Italy). When not specified, the chemicals and reagents were of the highest purity grade commercially available.

### 2.2. LN Preparation

LNs that were loaded with CPS were prepared by the solvent injection method [23]. Softisan© 100 (0.2 g) and drug (0.016 g; 0.026%) were dissolved at 35 °C in ethanol (10 mL) and then added, with continuous stirring at 500 rpm, by means of a Hei-Tec magnetic stirring hotplate (Heidolph Instruments, Schwabach, Germany), to a Tween 80 solution (50 mL) using a syringe that was equipped with a micro-needle. Stirring was continued overnight to allow for the complete evaporation of the organic solvent. After that, the LN dispersion was filtered and stored at 4 °C until use. Blank LNs were also prepared and characterized.

### 2.3. Determination of Drug Loading

The amount of CPS loaded into LNs has been determined using a Pellicon XL™ tangential ultrafiltration system (Millipore, Milan, Italy) that was equipped with a polyethersulfone Biomax 1000 membrane with a 1,000,000 molecular weight cut off (MWCO). The retained material was freeze-dried and after solubilization in dichloromethane and analyzed by HPLC using the method reported below. The calibration curves were performed on six solutions in the concentration range 10–100 µg/mL. The correlation coefficient was >0.99. Each point represents the average of three measurements and the error was calculated as standard deviation (±SD). CPS incorporation efficiency was expressed as drug recovery and calculated from:

Drug recovery (%) = (mass of drug in nanoparticles/mass of nanoparticles fed to the system) × 100

Possible lipid interferences during UV determination of CPS were also investigated by comparing the two standard curves of CPS alone and in the presence of lipids. The differences observed between the standard curves were within the experimental error, thus inferring that no lipid interference occurred (data not shown).

### 2.4. Particle Size Distribution 

The mean particle size of the lipid dispersions was measured by Photon Correlation Spectroscopy (PCS). Analyses were performed at 25 °C while using a Zetasizer Nano ZS (Malvern, UK; Laser 4 mW He–Ne, 633 nm, Laser attenuator Automatic, transmission 100–0.0003%, Detector Avalanche photodiode, Q.E. > 50% at 633 nm, T = 25 °C). The results were also expressed as intensity Distribution (Di), i.e., the size below which is placed the 10% [Di(10)], 50% [Di(50)], and 90% [Di(90)] of all the particles. 

All of the data are expressed as means of at least three determinations carried out for each preparation lot (three lots for each sample).

### 2.5. Atomic Force Microscopy (AFM)

The morphology of the samples was evaluated by AFM observations (Atomic Force Microscope, Park Instruments, Sunnyvale, CA, USA) at room temperature (about 20–25 °C) operating in air and in noncontact mode. Triangular silicon tips were used for this analysis. The resonant frequencies of this cantilever were found to be about 190 kHz. A drop of each sample was diluted with water (about 1:100 v/v) before being applied on a small mica disk (1 cm × 1 cm); after 2 min., the excess of water was removed using paper filter. “Height image” or “topographical image” was obtained from the record of changes in Z-axis of the piezo necessary to maintain the fixed oscillation amplitude and described the morphology and the macroscopical features of the sample. The “height profile” particles, which were obtained by elaborating the “height images” and reported in x/y graphics, contributed nicely to describing the samples. The images were flattened using second-order fitting to remove sample tilt. The mean size of LN was carried out by processing the AFM images with the ProScan Data Acquisition software that was developed under Windows. The sizes of the LN were determined disassembling the images in arbitrary lines that cross the small vesicles. We chose a line and limited each LN positioning two sliders to carry out the dimension. All of the data averaged measurements for each experiments.

### 2.6. Differential Scanning Calorimetry (DSC) Analysis

Thermograms of samples (raw materials, empty and loaded capsaicin lipid nanoparticles were carried out through Mettler DSC 12E that was equipped with a Haake thermocryostate model D8-G; each measurement was performed three times. The correlated software permitted data acquisition. The analyses were performed at a speed of 5 °C/min. in the range from 25 °C to 100 °C after calibration of the equipment.

### 2.7. In Vitro Release Study

Franz diffusion cells (LGA, Berkeley, CA) with an exposed surface area of 0.75 cm^2^ and a receiver compartment volume of 4.5 mL were used to determine the release profile of pure CPS and CPS-LN. The receptor compartment was filled with a water–ethanol solution (50:50) to create the sink condition, since CPS has a limited solubility in buffer. The solution has been stirred at 500 rpm and thermostated at 32 ± 1 °C during all of the experiments [24]. The testing samples (350 µL) were placed in the donor cell maintaining a complete and intimate contact with the surface of a cellulose acetate membrane (0.2 µm pore size, 25 mm diameter, Sartorius; Göttingen, Germany). Each experiment was run in duplicate for 24 h. At predetermined intervals, samples (200 µL) of receiving solution were withdrawn and replaced with fresh solution. The samples were analyzed for drug content by High Performance Lipid Chromatography (HPLC), as described below.

### 2.8. In Vivo Study

#### 2.8.1. Animals 

Male CD1 mice aged between eight and nine weeks were used in the present study. The institutional animal care and use committee approved all of the procedures (IACUC, ex art. 9 DL116-92, 9 September 2005). The animals were maintained on a 12-h light/dark cycle and allowed free access to food and water. For behavioral experiments, mice were acclimated to the experimental room one hour before testing. A researcher that was blind to the pharmacological treatment performed all of the experimental procedures and tests.

#### 2.8.2. In Vivo Administration

For the evaluation of spontaneous pain, ten microliters of a plain drug solution (CPS-STD) or an LNs dispersion (CPS-LN) or the respective vehicles (VEH-STD and VEH-LN) were administered by subcutaneous injection.

CPS-STD was formulated while using Tween 80 (10% *w*/*w*), ethanol (10% *w*/*w*), CPS (0.026% *w*/*w*), and distilled water (79.97% *w*/*w*). Subcutaneous injection was performed in the right hind paw (intraplantar administration, i.pl.) of the mouse immediately before the behavioral evaluation. 

#### 2.8.3. Spontaneous Pain

For the evaluation of spontaneous pain, the CD1 mice were placed in a transparent cage immediately before the i.pl. injection of the two different drug formulations (CPS-STD and CPS-LN) or the respective vehicles (VEH-STD and VEH-LN). The time (seconds) spent in nociceptive behavior (paw liking, lifting, and shaking) was measured for up to five minutes after the injection by a researcher that was blind to the treatment of the animals. 

#### 2.8.4. Preparation of Protein Extracts from Mouse Skin 

After seven days from i.pl injection, the animals were sacrificed and skin biopsies that formed the site of injection were removed. Skin homogenates were obtained, as previously described [25]. Protein concentration was measured by using the Bio-Rad Dc Protein Assay kit according to the manufacturer’s protocol.

#### 2.8.5. Western Blot Analysis

For the western blot analysis of the TRPV1 expression, ten μg of total protein extracted form skin biopsies were separated by SDS-PAGE (Sodium Dodecyl Sulphate–polyacrylamide gel electrophoresis) and transferred onto nitrocellulose membranes for antibody detection (Criterion blotter; Bio-Rad Laboratories, Hercules, CA, USA). The nitrocellulose membranes were blocked at room temperature in Odyssey blocker (LI-COR Biosciences, Lincoln, NE) for 1 h, and then incubated with the following primary antibodies: anti-TRPV1 polyclonal antibody (1:1000, Santa Cruz); anti-actin monoclonal antibody (1:1000, Sigma) over night at 4 °C. Secondary antibodies were: goat anti-rabbit (IRD800CW) and goat anti-mouse (Alexa 680, LI-COR, Bioscience) antibodies, being concomitantly incubated for 1 h at room temperature. The proteins were detected and quantified with the Odyssey Infrared Fluorescence Imaging System (LI-COR). The values were expressed as integrated intensity. 

### 2.9. HPLC Analysis

The HPLC apparatus consisted of a Shimadzu LC10 AT Vp (Milan, Italy) that was equipped with a 20 µL loop injector and a SPD-M 10 A Vp Shimadzu photodiode array UV detector. The HPLC method used for CPS determination was a slight modification of a method that was reported in literature [26]. In particular, the chromatography was performed using a Symmetry Shield Waters C18 RP column (particle size, 5 µm; 25 cm × 4.6 mm i.d.; Waters S.P.A, Italy). The chromatographic method provided a mobile phase that was composed of acetonitrile (80%) and a water solution (20%) containing 1% acetic acid. The flow rate was set at 1 mL/min. and the detection was effected at 284 nm, while the CPS retention time was 8.4 min.

### 2.10. Statistical Analysis

Statistical analysis of in vivo data was performed while using the one-way ANOVA, followed by two different post hoc tests: the Bonferroni’s Multiple Comparison test for the determination of the spontaneous pain due to CPS i.pl. injection and the Fisher’s test to determine the TRPV1 skin expression.

## 3. Results and Discussion

### 3.1. LN Formulation and Characterization

The solvent injection method that was used in this work appeared to be suitable for LN preparation. CPS-LN were prepared with Softisan©100 (Hydrogenated Coco-Glycerides) as lipid and Tween 80 as surfactant. We decided to use this material after a lipid screening for the identification of matrices for CPS incorporation. Particularly, we chose hydrogenated Coco-Glycerides to prepare lipid nanoparticles, since, in preliminary studies, it showed a high affinity toward the active. This evidence justifies the high encapsulation efficiency found for CPS-LN formulation (97.3 ± 2.53%). 

Table 1 reports PCS analysis. Free LNs were characterized by particles with diameters ranging about 250–300 nm and with a polydispersity index (PDI) of about 0.3.

When considering the PDI values, which correctly describe the size distribution of the samples, CPS-LN showed the same median diameter Di(50) of unloaded LN (about 340 nm), but a greater presence of a population of LN with diameter proximally to 1 um (30% vs. 2% of empty LN), with the evidence of a marked increase in sample complexity. 

**Figure 1:**
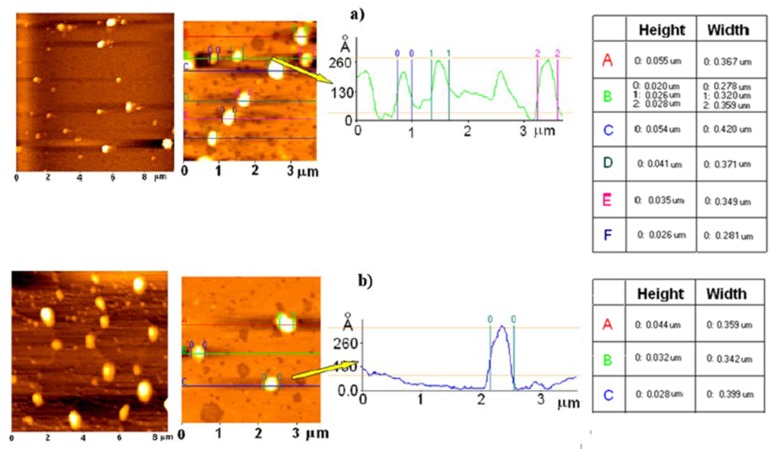
Atomic Force Microscopy (AFM) images, height profile and elaboration of (**a**) unloaded LN and (**b**) Capsaicin loaded lipid nanocarriers (CPS-LN).

These results were confirmed by AFM analysis on mica, as reported in Figure 1. All of the samples presented nanoparticles with a spherical shape and well-defined contours. AFM “height images” (scansion of about eight-micron) described the major heterogeneity of CPS-LN if compared to unloaded LN, even if particles that were sized from 200 to 400 nm remained representative of this sample. 

The xy graphics describing the “height” profile of LN (Figure 1a) nicely contributed to describing the regular shape (symmetry) of the LN, also after CPS encapsulation (Figure 1b).

Moreover, the LN can be described as flattened-platelet shaped carriers since the diameters of LN were higher than the related heights with a height (H)/width (W) ratio close to 1/10 [27]. In agreement with the studies that were reported in literature, the difference between width and height could be partially due to the interaction between the sample and the substrate, as well as to the continuous movement of the cantilever probe of the AFM that, by pushing and warming the particles, might determinate a deformation of their original morphology [28]. 

**Figure 2:**
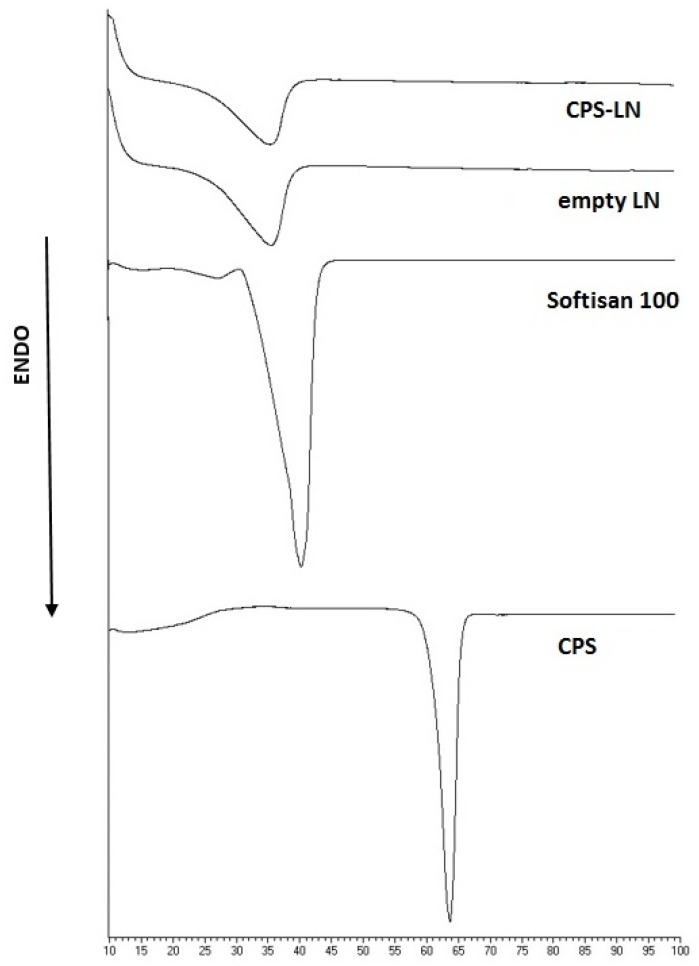
Differential Scanning Calorimetry (DSC) thermogram scans of components, unloaded and CPS-LN.

The physical state of lipids affects the permeability and the stability of the LN. DSC studies provide insight into the phase transition in LN that is responsible for the change in physical state of the system. DSC analysis characterized unloaded and loaded LN. Figure 2 shows the DSC thermograms of the component of unloaded LN and CPS-LN.

The calorimetric curves of raw materials showed a melting point of 39 °C for Softisan© 100 and 65 °C for CPS, respectively. Unloaded LN and CPS-LN both showed a slight decrease of melting point correlated to lipid constituent (36.2 °C for LN and 36.5 for CPS-LN) and a modification of the endothermic peak that appeared broader. The depression of endothermic peak is often observed when the bulk lipid is transformed into LN. 

### 3.2. In vitro Release Study

**Figure 3:**
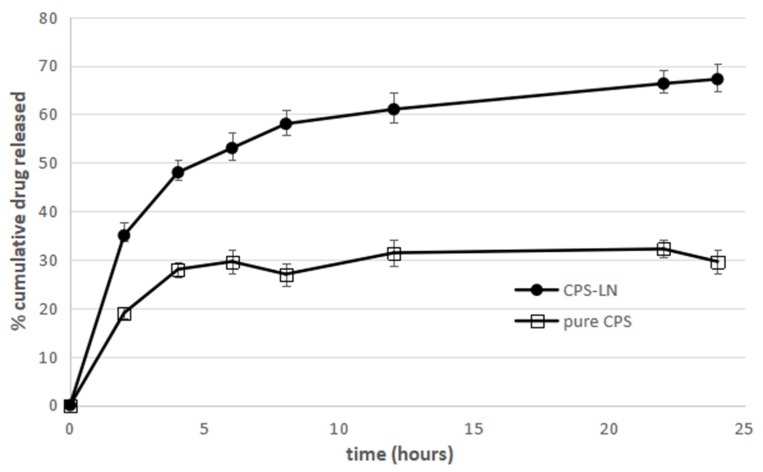
Percentage of cumulative CPS released from pure CPS and CPS-LN. Each point represents the mean value ± S.D. (n = 3).

The CPS release profile from CPS-LN (Figure 3) exhibits a minimum burst effect within the first 2–3 h. CPS-LN continued to release its content until the 24th hour, at which it released 70% of encapsulated drug, exhibiting a steep increase in the amount of the released CAP when compared to that of pure CAP (Figure 3). 

Therefore, it could be concluded that drug incorporation in CPS-LN can prolong drug release, owing to the time that is required for the drug to diffuse into the release medium from LN matrix. This result is consistent with the findings of other researchers regarding drug release from lipid nanoparticles [29,30,31].

### 3.3. In Vivo Study

**Figure 4:**
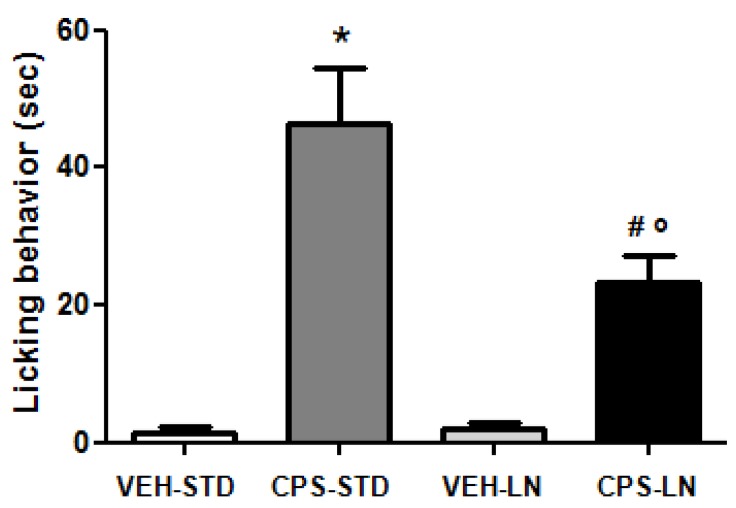
Intraplantar (i.pl.) injection of CPS induces spontaneous pain. The nocifensive response to i.pl. injection of CPS (0.125%/10 μL) dissolved in standard vehicle or included in LN in naïve mice is shown. Data are means ± S.E.M of 6 mice, and refer to the number of sec spent in licking behavior in the first 5 min. following injection. * *p* < 0.05 vs. mice injected with vehicle, # *p* < 0.05 vs. mice injected with LN, ^o^ *p* < 0.05 vs. mice injected with capsaicin (one-way ANOVA followed by “Bonferroni’s Multiple Comparison Test”).

The acute activation of TRPV1 receptors by CPS is known to lower pain threshold [32]. In this experiment, CPS was locally administrated in the plantar surface or the right hind-paw. The intradermal injection of CPS was selected to rapidly deposit a precise amount of drug directly into the site of injection. This procedure is known to produce a sensation of intense pain at the site of injection [33]. In our experiments, spontaneous pain that is induced by intraplantar injection of CPS has been evaluated in mice for five minutes after administration and measured as liking, lifting, and shaking behavior of the injected paw (Figure 4).

As expected, the activation of TRPV1 receptors by both CPS formulations tested induces spontaneous pain. The inclusion of CPS in LN-lipid matrix induced a lower pain response when compared to drug dissolved in a standard vehicle (CPS-STD), according to western blot results. No differences in pain behavior after VEH-STD and VEH-LN administration were observed (Figure 4), showing that neither the standard vehicle nor the unloaded LN-lipid matrix induce a pain response per se. Thus, the nociceptive behavior observed in CPS-STD and CPS-LN groups has to be related to the presence of CPS that is able to activate TRPV1 receptors. Moreover, no pain response was observed in the contralateral uninjected paw (data not shown), thus excluding the systemic effect of the drug. 

### 3.4. TRPV1 Skin Expression

**Figure 5:**
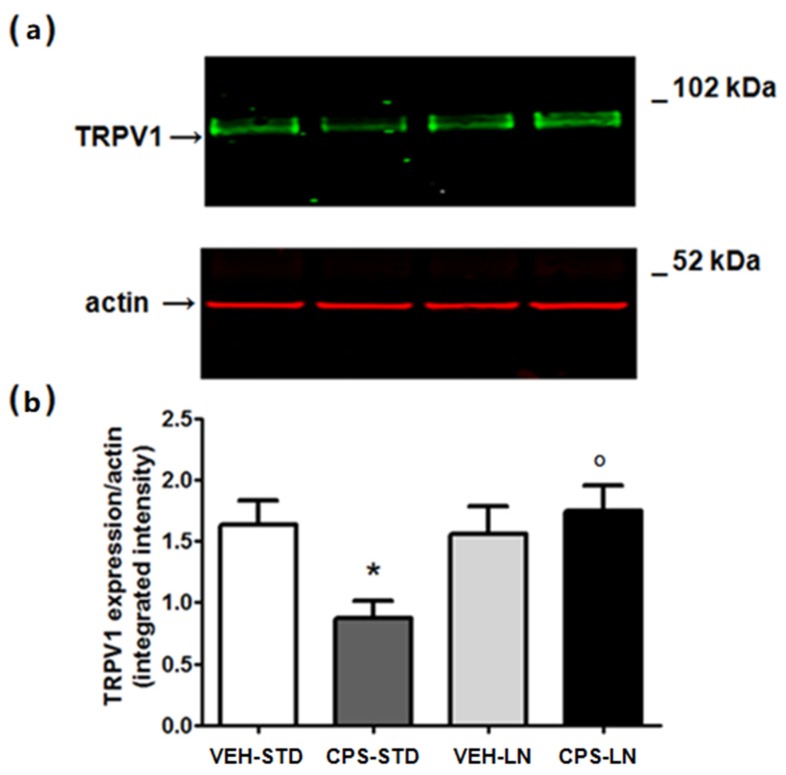
Expression of TRPV1 receptor in the skin of CD1 mice after seven days from the i.pl. injection of CPS included in standard vehicle or LN. I.pl. injection of CPS induces a significative downregulation of TRPV1 receptors that is blocked by LN inclusion. A representative immunoblot of TRPV1 in the skin of naïve mice and mice injected with capsaicin (0.125%/10 μL) in the absence or presence of LN is shown in (**a**). Densitometric analysis is shown in (**b**), where values are means + S.E.M. of four determinations. * *p* < 0.05 vs. vehicle mice; ^o^ *p* < 0.05 vs. mice injected with capsaicin (one-way ANOVA followed by Fisher’s post hoc test).

Prolonged exposure to CPS can desensitize the TRPV1 receptor or induce its internalization and degradation [14,15]. Consistent with data from the literature, the local i.pl. injection of CPS-STD induced a significant reduction of TRPV1 expression in the mouse skin at the site of injection, as shown in Figure 5. Interestingly enough, CPS inclusion in LN prevented from TRPV1 down-regulation in the skin, an effect that is probably due to a slow release of the drug, thus preventing TRPV1 internalization and degradation.

Agonist-induced defunctionalization of TRPV1 receptors is the underlying mechanism for long-term nociceptors desensitization. CPS-induced down regulation of membrane TRPV1 levels has been used as a strategy for pain treatment in a variety of formulations, such as creams, lotions, or high concentration capsaicin patches [12,13]. However, different functions of TRPV1 receptors other that pain perceptions have emerged. For example, a number of studies indicate a role for TRPV1 receptors in carcinogenesis [34,35,36].

Some studies indicate that TRPV1 receptor antagonist promote skin carcinogenesis through the EGFR, thus a role for TRPV1 receptors as tumor suppressors have been proposed [37]. This section might be divided by subheadings. It should provide a concise and precise description of the experimental results, their interpretation, as well as the experimental conclusions that can be drawn.

## 4. Conclusions

This study provides important evidence regarding the efficacy of LNs as carriers for CPS administration.

CPS-LNs were formulated by using the solvent injection method, which appeared to be suitable for LN preparation. In fact, the particles were in the nanometric range, showing good homogeneity and high encapsulation efficiency. The AFM study revealed a regular shape of LN, also after CPS encapsulation.

The in vivo study pointed out that CPS inclusion in LN-lipid matrix induced a lower pain response when compared to drug dissolved in a standard vehicle (CPS-STD).

The lack of TRPV1 receptor down regulation in the site of CPS-LN application might be due to a slower release of CPS from the lipid matrix that could prevent TRPV1 internalization and degradation. Thus, while the agonist-induced TRPV1 reduction in the intra-epidermal nerve fibers is useful for inducing long-term nociceptors desensitization for pain relief, the chronic blockade or the absence of TRPV1 receptors might increase the risk of tumorigenesis. With this in mind, we studied a CPS-containing formulation able to provide long-term activation of TRPV1 receptors without the unwanted reduction of TRPV1 receptor expression.

In conclusion, LNs could be useful as a valid alternative to conventional vehicles when a controlled release of CPS is required to prevent TRPV1 defunctionalization after prolonged activation of the receptor.

## Figures and Tables

**Table 1:** Particle size, intensity distribution (Di), and polydispersity index (PDI) of LN based formulations.

Sample	Z Average (nm)	PDI	Peak1 (nm)	(%)	Peak2 (nm)	(%)	Di(10) (nm)	Di(50) (nm)	Di(90) (nm)
empty LN	296 ± 26	0.282 ± 0.007	356 ± 51	98 ± 50	1420 ± 14	1.6 ± 2	64 ± 23	330 ± 43	1112 ± 58
CPS-LN	287 ± 86	0.410 ± 0.004	387 ± 30	76 ± 15	876 ± 54	28 ± 2	112 ± 32	342 ± 72	780 ± 68
